# Investigation Into the Impact of Solvents on the Phytochemical Composition, Antioxidant Capacities, and Antihyperglycemic Activities of *Erigeron annuus* (L.) Pers.

**DOI:** 10.1155/bmri/6650124

**Published:** 2025-04-15

**Authors:** Mehak Thakur, Rachna Verma, Dinesh Kumar, Manickam Sivakumar, Tabarak Malik

**Affiliations:** ^1^School of Biological and Environmental Sciences, Shoolini University of Biotechnology and Management Sciences, Solan, India; ^2^Department of Chemistry, Faculty of Science, University of Hradec Kralove, Hradec Kralove, Czech Republic; ^3^Centre for Advance Innovation Technologies, VSB-Technical University of Ostrava, Ostrava, Czech Republic; ^4^School of Bioengineering and Food Technology, Shoolini University of Biotechnology and Management Sciences, Solan, India; ^5^Petroleum and Chemical Engineering Department, Faculty of Engineering, Universiti Teknologi Brunei, Bandar Seri Begawan, Brunei Darussalam; ^6^Department of Biomedical Science, Institute of Health, Jimma University, Jimma, Oromia Region, Ethiopia; ^7^Division of Research and Development, Lovely Professional University, Phagwara, Punjab, India

**Keywords:** anti-inflammatory, anti-oxidant, cytotoxicity, *Erigeron annuus*, phytochemical

## Abstract

This study aims to assess the phytochemical composition, antioxidant potential, and antidiabetic properties of *Erigeron annuus* (L.) Pers. The ethyl acetate fraction of *Erigeron annuus* leaves exhibited the highest extraction rate (22.42%). The preliminary qualitative phytochemical analysis in crude extract and fractions is often performed using chemical tests. For quantitative analysis, spectrophotometric methods are widely used to estimate the concentration of phytochemicals. The antioxidant properties were evaluated using the 2,2-diphenyl-1-picrylhydrazyl (DPPH) radical scavenging assay and the ferric reducing antioxidant power (FRAP) assay, which measures the reduction of Fe^3+^ to Fe^2+^. Qualitative screening revealed the presence of tannins, flavonoids, phenols, saponins, and alkaloids. Notably, the ethyl acetate fraction showed significantly (*p* < 0.05) higher total phenolic content (70.01 ± 1.1 mg/g) and total flavonoid content (80.29 ± 1.03 mg/g). This fraction also demonstrated substantial *α*-amylase inhibitory activity and antioxidant potential, suggesting the ability of polyphenols to reduce *α*-amylase activity. The *α*-amylase inhibition (23.15 ± 1.22% to 67.31 ± 2.01%) activity and IC_50_ value (40.59 ± 0.03*  μ*g/mL) were notably higher in the ethyl acetate fraction compared with the standard drug metformin (19.88 ± 1.51*  μ*g/mL). *Erigeron annuus* ethyl acetate fraction exhibited significantly higher glucose levels (10.88% ± 1.29% to 65.11 ± 0.94%) and conducted a lipid peroxidation experiment utilizing egg yolk as the source of lipids with high content. The most bioactive fraction was evaluated for cytotoxicity against the HEK293 cell line. The cytotoxicity assay revealed that 50% cell viability was observed at a concentration of 50 *μ*g/mL, indicating that the plant extract is nontoxic at concentrations below this threshold. Furthermore, the dominant fraction was further investigated using liquid chromatography–mass spectroscopy and high-performance thin-layer chromatography techniques from the selected plant. Moreover, an in vivo study will be performed to evaluate the antidiabetic efficacy of *Erigeron annuus*, isolate and characterize its bioactive components, and examine its molecular mechanism of action to improve its therapeutic applicability.

## 1. Introduction

Medicinal plants are used by 80%–85% of the global population for basic healthcare. Medicinal plants serve as the cornerstone of traditional medicine, contributing significantly to the development of modern medications and their derivatives [1]. Plant-based antioxidants particularly polyphenols have garnered substantial interest due to their potential medical benefits [2]. Extracts or active components derived from medicinal plants are commonly employed in traditional medicine practices [3]. Currently, over 1000 plants have demonstrated efficacy in treating diabetes mellitus.

Many pharmacological drugs derived from medicinal plants either directly extracted or indirectly synthesized [4]. Several herbal medications for treating diabetes includes *Allium sativum* L.*, Aloe vera* Mill.*, Coccinia indica* (L.) Voigt*, Eugenia jambolana* (L.) Skeels*, Mordica charantia* L., *Ocimum sanctum* L., *Trigonella foenum*-graecum L., *Erigeron annuus* (L.) Pers.*, Fagopyrum esculentum* Moench, and *Vernonia amygdalina* Del. [5].

Natural antioxidants from plants, like phytochemical compounds and their derivatives or crude extracts (CEs), slow the cell-damaging effects of oxidative stress. Any disruption or deficiency in the antioxidant process can result in oxidative stress, contributing to various diseases [6].

Diabetes mellitus (DM) is a condition characterized by a disruption in metabolism characterized by persistent hyperglycemia or hypoglycemia, accompanied by changes in carbohydrate, lipid, and protein metabolisms, leading to insulin deficiency or elevated insulin levels [7]. Epidemiological data indicates that there are 451 million adults worldwide aged 18–99 with DM, which is projected to increase to 693 million by 2045 [8]. In 2017, approximately 5 million global fatalities among individuals aged 20–99 was attributed to diabetes [9]. In India, an estimated 1.3 million people aged 20–79 are expected to have diabetes, with a prevalence of 2.9% by 2030. Current therapeutic approaches often have various adverse effects that adversely impact the patient's quality of life. Consequently, the significance of plant-based therapy is on the rise, and plant-based alternative medicine has flourished over time [10].

Several new bioactive compounds derived from plants that lower blood sugar levels [11]. Metformin, commonly known as glucophage, is an oral antidiabetic drug derived from *Gelga officinalis* L [1]. These medications have been shown to provide antidiabetic and therapeutic benefits [12]. The antidiabetic effects of medicinal plants operate through various mechanisms, such as inhibition of *β*-galactosidase, *α*-glucosidase, prevention of oxidative stress, pancreatic *β*-cell dysfunction, and including activation of glycogenesis [13, 14]. The antioxidant action assists in preserving *β*-cell activity in individuals with diabetes [15]. The pathophysiology of DM and its consequences cause oxidative stress. Several studies have emphasized the significance of antioxidants in treating diabetes and its consequences [16]. Natural products have a vital function in the search for novel medicinal agents and have garnered significant interest as sources of bioactive compounds [17]. *Erigeron annuus* belongs to the Asteraceae family, and India is home to approximately 18,664 species of higher plants, and about 900 of them are classified under the Asteraceae family [18]. *E. annuus*, a daisy-like plant, grows organically and has been traditionally used for various purposes in Korea, Japan, and China, such as treating bronchitis, cough, and convulsions. In traditional Eastern medicine, *E. annuus* addresses dyspepsia, epidemic hepatitis, enteritis, lymphadenitis, and hematuria [19]. Flavonoids are class of polyphenolic chemicals with a benzo-*γ*-pyrone structure. Phytochemical analysis has identified *γ*-pyranone derivatives such as 2-pyrone, 4-pyrone, flavonoids, and triterpenoids present in this plant. These derivatives are commonly found in natural products such as flavonoids and other polyphenolic chemicals and can have a variety of biological functions, including antioxidant, anti-inflammatory, and antidiabetic effects [20]. Several studies highlighted the pharmacological properties of *E. annuus* and its constituents have antiatherosclerotic, antiproliferative, antiprotein glycation, and antioxidant properties [21, 22]. The pharmacological properties of plants that can treat diabetes are commonly associated with a range of components, including phenolic substances (such as anthraquinones, physcion, C-glycosylated anthrones, and 2-hydroxy-3-methyl-anthraquinone), flavonoids, glycosides, terpenoids, lipids steroids, alkaloids, peptides, and other substances [23], the specific antidiabetic action of the *E. annuus* plant has not yet been reported.

Furthermore, phenolic glycosides have been linked to many biological characteristics including antioxidant, antidiabetic, and anti-inflammatory activities [24]. Notably, there is an insufficient of literature reports examining the use of *E. annuus* leaves for screening antioxidant, anti-inflammatory, *α*-amylase, glucose uptake, and lipid peroxidation activities. Hence, due to the plentiful presence of phenolic glycosides and other phenolic compounds, this work aims to find potential biological fractions obtained from the leaves of *E. annuus.* High-performance thin-layer chromatography (HPTLC) was employed to identify the main phytochemicals in the most bioactive fraction. The findings of this study are expected to contribute to identifying new bioactive compounds, particularly antioxidant, and antidiabetic compounds, within the active fraction. These findings may hold promise for further exploration in developing an effective drug system for diabetes.

## 2. Materials and Methods

### 2.1. Identification of a Plant Species

**Figure 1:**
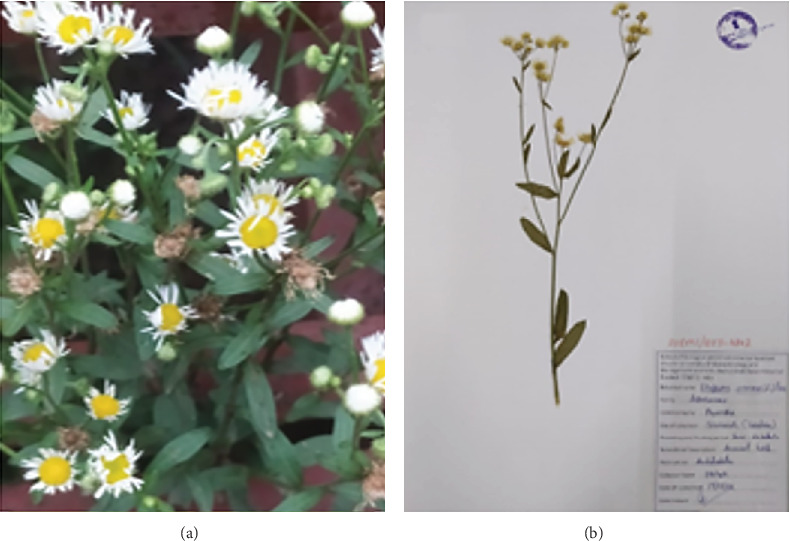
(a) Plant *E. annuus*. (b) Herbarium sheet of *E. annuus.*

The chosen medicinal plant was collected from June to August 2022. Subsequently, *E. annuus* leaves were collected from Sirmaur district of Himachal Pradesh located at GPS coordinates 30° 45⁣′ 0⁣^″^ N, 77° 30⁣′ 0⁣^″^ E and authenticated at the Botanical Survey of India, Nauni, with the assigned accession number (Acc-00041) and voucher number as shown in Figure 1 (SUBMS/BOT-4842). The collection was conducted in the wild conditions. The plant identification was carried out by Dr. Kumar Ambrish, a scientist at Dr. Y.S. Parmar University, Nauni Campus, Solan-173230, Himachal Pradesh, India. The leaves of *E. annuus* the specimens were conveyed to the laboratory in plastic bags for subsequent examination.

### 2.2. Preparation of Methanolic Crude Extracts (MCEs)

The coarse leaf powder (20 g) was subjected to extraction with methanol (200 mL) using an orbital shaker set at 40°C for 72 h, following the procedures outlined by Ghosh and Chandra [24]. Separately, the concentrated MCEs from the leaves were obtained and stored at 4°C. The filtrate was then strained, then filtered the solution using Whatman filter paper no. 1, and then concentrated it in a hot air oven at 37°C. The resulting concentrated CEs from the leaves were collected individually and preserved at 4°C.

#### 2.2.1. Fractionation of MCEs

The bio-guided fractionation followed the protocol outlined by Kumar et al. [25]. Specifically, 5 g of *E. annuus* leaves were individually dissolved in 100 mL of distilled water with agitation for 10 min. Afterward, the mixture was defatted with 100 mL of petroleum ether. After separation, the upper petroleum ether layer in the separating funnel, containing nonpolar lipophilic impurities, was discarded. The lower extract layer was retained for further analysis and sequentially partitioned using a separating funnel. First, an equal volume of chloroform (CF) was added, and the lower layer was collected as the CF fraction. Next, ethyl acetate was added to the remaining upper layer, and the upper ethyl acetate fraction (EAF) was collected. The final lower layer was retained as the aqueous residual fraction (AF). Each fraction (CF, EAF, and AF) was individually collected, dried, and kept at a temperature of 4°C in the refrigerator for future use. Passing through Whatman filter paper no. 1 and being concentrated at 37°C in a hot air oven.

### 2.3. Preliminary Qualitative Phytochemical Analysis

Various secondary metabolites, such as saponins, flavonoids, alkaloids, tannins, and phenols, were evaluated in all solvents following the procedures described by Shaikh and Patil [26]. Standard compounds, including quercetin for flavonoids, diosgenin for saponins, caffeine for alkaloids, gallic acid for phenols, tannic acid for tannins, and linalool for terpenoids, were used as standards.

#### 2.3.1. Test for Phenols

Ferric chloride test: For 10 mg of CEs and fractions, a few drops of ferric chloride solution were added. The appearance of a blueish-black color showed the presence of phenol.

#### 2.3.2. Test for Flavonoids

Sulfuric test: To the CEs and fractions, a few drops of H_2_SO_4_ were added. The appearance of orange color revealed the presence of flavonoids.

Lead acetate test: For a lead acetate test, extract and fractions were treated with a few drops of lead acetate solution. Formation of yellow precipitate shows the presence of flavonoids.

#### 2.3.3. Test for Tannins

Ferric chloride test: A low quantity of extract and fractions was combined with water and heated or boiled in the water bath. The solution was filtered, and the resulting filtrate was treated with ferric chloride. It resulted in dark green color, which indicated the presence of tannins.

#### 2.3.4. Test for Saponins

Foam test: 0.5 mg of extract and fractions was mixed with 5 mL of distilled water. The formation of foam indicated the presence of saponins (a foamy mist of small bubbles).

#### 2.3.5. Test for Terpenoids

Salkowski's test: Few drops of conc. H_2_SO_4_ were added to the extract and fractions (shaken well and allowed to stand). Golden yellow layer indicates the presence of terpenoids (at the bottom).

#### 2.3.6. Test for Alkaloids

Mayer's test: The appearance of cream-colored precipitates confirmed the existence of alkaloids when few drops were added to the plant extracts.

### 2.4. Qualitative Phytochemical Analysis Through Spectrophotometric Method

#### 2.4.1. Quantification of Total Phenolic Content

The total phenol concentration of plant extracts was determined using the Folin–Ciocalteu method, as described by Alam et al. [27]. The Folin–Ciocalteu reagent (5 mL) was carefully combined with 1 mg/mL samples. After a 10-min incubation period, we introduced 4 mL of a 7.5% solution of sodium carbonate (Na_2_CO_3_) and allowed the reaction to continue at room temperature for 1 h. The measurement of absorbance was conducted at a wavelength of 765 nm, and each sample was tested three times. A concurrent procedure was utilized to create a standard curve using gallic acid at concentrations ranging from 20 to 100 *μ*g/mL. The outcomes were then quantified and reported in milligrams of GAEs (gallic acid equivalents) per gram of extract.

#### 2.4.2. Quantification of Total Flavonoid Content (TFC)

The plant extract's TFC was measured using the colorimetric aluminum chloride technique defined by Bondonno et al. [28]. During this process, 1.5 mL of 95% ethanol was added and combined with the plant-based extract and fractions (0.5 mL and 1 mg/mL) for a duration of 10 min. Afterwards, a solution containing 0.1 mL of aluminum chloride (10%) and 1 M potassium acetate was introduced. The solution was diluted with distilled water to get the desired volume of 5 mL. After a comprehensive blending process, the mixture was allowed to rest at room temperature for a duration of 30 min. Quercetin was employed as a standard at concentrations ranging from 20 to 100 *μ*g/mL. The analysis was performed at a wavelength of 415 nm. The flavonoid contents in all tested extracts were quantified as milligrams of quercetin equivalents per gram of dry weight using the calibration curve. The blank employed a solution consisting of 10% aluminum chloride and distilled water, and this procedure was done three times.

#### 2.4.3. Quantification of Total Tannin Content

The tannin content was assessed using the approach established by Morsy [29]. In this technique, plant extract (0.5 mL) was treated with 1% potassium ferricyanide and 1% ferric chloride. The resultant combination was further diluted with distilled water to achieve a final volume of 10 mL. Similarly, a range of standard tannic acid solutions (5–25 *μ*g/mL) were made using the same method and were analyzed at a wavelength of 720 nm. The tannin concentration in CE and fractions was measured in milligrams of tannic acid equivalents per gram of dry weight.

#### 2.4.4. Quantification of Total Saponin Content

Goel et al. [30] used the vanillin–sulfuric acid colorimetric technique to determine the saponin concentration in CE and all fractions. This study used diosgenin as the standard. The plant extract (0.5 mL, 1 mg/mL) was treated with a solution of vanillin (0.5 mL, 8% ethanol) and concentrated sulfuric acid (5 mL, 72%). After 15 min of stirring in a water bath at 60°C, the solution was chilled for 10 min. Similarly, standard diosgenin solutions (5–60 *μ*g/mL) were generated following the same process. After chilling for 10 min, the absorbance at 560 nm of extract and standard solutions was measured to determine the saponin concentration.

#### 2.4.5. Quantification of Total Alkaloid Content

The bromocresol green (BCG) technique, as described by Ajanal et al. [31], was used to determine the total alkaloid content in CE and fractions. The samples were solubilized in 2 N hydrochloric acid (HCl) at a concentration of 1 mg/mL. Filtration with 0.1 N NaOH neutralized the pH of the phosphate buffer solution. Subsequently, 1 mL of the solution was introduced into a separating funnel, together with 5 mL of BCG and 5 mL of phosphate buffer. The chemical was vigorously stirred, and then, it was extracted using chloroform. The sample was created by combining and diluting it in a 50-mL volumetric flask using chloroform. Additionally, solutions of standard caffeic acid with concentrations ranging from 5 to 60 *μ*g/mL were prepared. The absorbance of the samples was measured at a wavelength of 470 nm. The experiment was replicated three times, employing accurate measurements of caffeine to evaluate the alkaloid content in the extracts (mg/g).

#### 2.4.6. Quantification of Total Terpenoid Content

The total terpenoid content was calculated using the technique described by Truong et al. [32], with minor adjustments, and linalool as the reference. This method involved treating a CE and fractions (1 mL, 1 mg/mL) with 2 mL of chloroform and 200 *μ*L of pure sulfuric acid. The mixture was left to settle at room temperature in the dark for 1.5–2 h, resulting in a reddish-brown precipitate. After 2 h, the supernatant from the reaction mixture was filtered and 3 mL of 95% methanol was added. The fluid was stirred until solid particles appeared. A similar process was used to create standard linalool solutions (5–25 *μ*g/mL), and the absorbance of both the test solution and the standard was measured at 538 nm.

### 2.5. Evaluation of the Antioxidant Activity of *E. annuus* CE and Fractions

#### 2.5.1. DPPH (2,2-Diphenyl-1-Picrylhydrazyl) Assay

The compound as a standard, ascorbic acid, were tested for their capacity to scavenge unstable DPPH radicals using the technique published by Hu et al. [33]. Initially, 3.94 mg of DPPH was combined with 100 mL of methanol. Then, DPPH solution (1 mL) was added to 3 mL samples at different concentrations (20–100 *μ*g/mL). In the control test, an equal volume of methanol was applied. The mixture was briskly mixed before resting for 30 min at room temperature. Ascorbic acid was used as a standard. Afterward, the absorbance was measured at 517 nm. The percentage inhibition (I%) of the DPPH radical was computed using the Equation (1):

(1) $$\begin{array}{c} \%=\frac{A_{0}-A_{\text{s}}}{A_{0}}\times 100, \end{array}$$

where *A*_s_ is the sample absorbance and *A*_0_ is the control absorbance (all reagents except the sample). The inhibition percentages (I%) were plotted against the sample concentrations to analyze the antioxidant activity.

#### 2.5.2. Ferric Reducing Antioxidant Power (FRAP) Assay

The antioxidant's capacity to reduce ferric ions was tested using the methods provided by Benzie and Strain [34]. Samples were generated with varying doses (20–100 *μ*g/mL). Low pH conditions transform ferric ions and the 2,3,5-triphenyl-1,3,4-triaza-2-azoniacyclopenta-1,4-diene chloride complex (TPTZ) into ferrous. Absorption at 593 nm was then measured. A 0.02 M FeCl_3_ solution was prepared by dissolving 1 mL of 1 M HCL in 50 mL of distilled water. A FRAP reagent was prepared by mixing 300 mM CH_3_COONa buffer, 10.0 mM TPTZ solution, and 20.0 mM FeCl_3_ solution in a 10:1:1 ratio at pH 3.6. Ferrous sulfate served as a standard, and its absorbance at 593 nm was measured after 30 min at 37°C. The sample's antioxidant potential was evaluated using the linear ferrous sulfate normal calibration curve (*y* = *mx* + *b*), which depicts the connection between antioxidant concentration and ferric reducing ability at 1 *μ*M FeSO_4_. In this application, *y* generally denotes the concentration of antioxidants in mol Fe (II) equivalent per gram of material. The linear ferrous sulfate standard calibration curve calculates the parameters *m* and *b*, enabling the quantification of antioxidant ability in terms of Fe (II) equivalents [27].

### 2.6. *In Vitro* Antidiabetic Activities of *E. annuus* Extract and Fractions

#### 2.6.1. Determination of Lipid Peroxidation

The test was run at different concentrations (20–100 *μ*g/mL), following Das et al. [35] technique with minor changes. Initially, 1 mL of egg homogenate (0.5 mL at 10% v/v) and 0.1 mL of the CE and fractions were diluted in distilled water. To induce lipid peroxidation, 0.05 mL of FeSO_4_ (0.07 M) was added and incubated for 30 min. Following that, 1.5 mL of 20% acetic acid (pH adjusted to 3.5 with NaOH), 1.5 mL of 0.8% (w/v) thiobarbituric acid (TBA) in 1.1% sodium dodecyl sulfate, and 0.05 mL of 20% trichloroacetic acid were added to the mixture, which was rapidly stirred. The mixture was then heated to 95°C for 40 min. Following chilling, each test tube received 5.0 mL of butan-1-ol and was centrifuged for 10 min at 3000 rpm. The organic top layer's absorbance was measured at 532 nm. Ascorbic acid acted as a positive control. The capacity to inhibit lipid peroxidation was calculated as a percentage using Equation (2):

(2) $$\begin{array}{c} I\%=\frac{A_{\text{c}}-A_{\text{s}}}{A_{\text{c}}}\times 100, \end{array}$$

where *A*_c_ represents the optical density of the control, whereas *A*_s_ represents the optical density of the sample.

#### 2.6.2. Determination of Glucose Uptake

The glucose absorption was measured using the procedures published by Nair et al. [36]. Samples were generated with varying concentrations (30–300 *μ*g/mL). Then, 1 mL of the extract was then put on a glucose-filled dialysis membrane. The membrane's ends were tied with thread and immersed in a beaker containing 40 mL of 0.15 M sodium chloride and 10 mL of distilled water. The control solution contained 1 mL of 0.15 M sodium chloride, 22 mM glucose, and clean water. The beakers were then kept at room temperature on an orbital shaker. Every half hour, the amount of glucose flowing into the external solution was monitored. To halt the reaction, 1 mL of dinitro salicylic acid (DNSA) color reagent was added. Metformin was used as a standard drug. The percentage of inhibition was estimated using Equation (3):

(3) $$\begin{array}{c} I\%=\frac{A_{\text{o}}-A_{\text{s}}}{A_{\text{o}}}\times 100 \end{array}$$

where *A*_o_ represents the absorbance of the control, whereas *A*_s_ represents the absorbance of the sample.

#### 2.6.3. Determination of Protein Denaturation

A changed version of the bovine serum albumin (BSA) test, which was first described by Deruaz et al. [37], to check how well crude and fractionated plant extracts from *E. annuus* could reduce inflammation. As a standard drug, diclofenac sodium was used, and test samples were made with *E. annuus* extract at concentrations ranging from 20 to 100 *μ*g/mL. BSA was dissolved in 15 mL of deionized water, yielding a concentration of 0.4% (w/v). The pH of the solution was raised to 7.6 at 25°C using tris-buffered saline, with a final concentration of 0.05 M tris and 0.15 M sodium chloride. Methanol was used to dissolve the plant extract, creating a stock solution with a concentration of 50 *μ*g/mL, or 0.005% w/v. The procedure for testing the positive (diclofenac sodium) and negative (methanol) controls was the same. The solutions were then subjected to a 10-min water bath heating at 72°C and a 20-min cooling period under controlled laboratory conditions. Spectrophotometer readings were taken at 660 nm to measure the turbidity of the solutions, indicating the degree of protein precipitation. The % inhibition of precipitation (protein denaturation) relative to the negative control was determined using Equation (4).

(4) $$\begin{array}{c} I\%=1-\frac{V_{t}}{V_{c}}\times 100, \end{array}$$

where *V*_*t*_ represents the optical density of the test sample and *V*_*c*_ represents the absorbance of the control. The concentration of the plant extract required to achieve 50% inhibition (IC_50_) was calculated using a dose–response curve.

#### 2.6.4. *α*-Amylase Inhibition Activity

The suppression of *α*-amylase was assessed using the experimental procedure outlined by Kumar et al. [38]. Five distinct concentrations (20–500 *μ*L) of the CE and fractions were obtained by diluting it with double-distilled water. It was mixed with 500 *μ*L of 0.02 M sodium phosphate buffer (pH 6.9) and 0.5 mg/mLof *α*-amylase solution. The mixture was then left to sit at 20°C for 10 min. Once the preincubation phase was over, 500 *μ*L of a 1% starch solution in sodium phosphate buffer (pH 6.9) with 0.006 M sodium chloride was added to each tube every 5 s. Subsequently, the reaction mixture was transferred to an incubator set at a temperature of 25°C and left undisturbed for 10 min. Then, 1 mL of DNSA color reagent was added to halt the process. The test tubes were immersed in a water bath that was heated to its boiling point for a duration of 5 min and thereafter allowed to cool down to the surrounding temperature. Metformin was used as a positive control. Afterwards, the reaction mixture was additionally diluted with 10 mL of distilled water, and the absorbance was determined at a wavelength of 540 nm. The inhibition was determined by employing Equation (5):

(5) $$\begin{array}{c} I\%=\frac{A_{o}-A_{s}}{A_{o}}\times 100, \end{array}$$

where *A*_*o*_ is the control absorbance and *A*_*s*_ is test sample absorbance.

### 2.7. *In Vitro* Antiproliferative Activity Against HEK293 Cell Line in Most Bioactive Fraction (EAF) of *E. annuus*

Cytotoxicity of the provided samples on HEK293 (procured from NCCS Pune) cell line was determined by 3-(4,5-dimethylthiazol-2-yl)-2,5-diphenyltetrazolium bromide (MTT) assay using methodology given by Fotakis and Timbrell [39]. The cells (10,000 cells/well) were cultured in 96-well plate for 24 h in MEM medium (minimum essential medium—AT154-1L-HIMEDIA) supplemented with 10% FBS (fetal bovine serum—HIMEDIA-RM 10432) and 1% antibiotic solution (penicillin-streptomycin-sigma-aldrich P0781) at 37°C with 5% CO_2_. Next day, cells were treated from different concentrations of the samples. Stock solution of samples was prepared in dimethyl sulfoxide (DMSO) and further diluted to get different concentrations in incomplete cell culture medium (without FBS). After incubation for 24 h, MTT solution (5 mg/mL) was added to cell culture and further incubated for 2 h. Cells without treatment were considered as control, and cells without MTT were considered as blank. At the end of the experiment, culture supernatant was removed, and cell layer matrix was dissolved in 100 *μ*L DMSO (DMSO–SRL-Cat no.-67685) and read in an ELISA plate reader (iMark, Biorad, USA) at 540 nm. The % viable cells were calculated by using following formula given below.

 $$\begin{array}{c} \%\text{Viable cells}=\left(\frac{A_{\text{test}}}{A_{\text{Control}}}\right)*100, \end{array}$$

where *A*_test_ is the absorbance of test sample and *A*_Control_ is the absorbance of control.

### 2.8. Detailed Quantification of Phytoconstituents in the Most Bioactive EAF of *E. annuus* Using Advanced HPTLC and LC-MS Techniques

#### 2.8.1. LC-MS Analysis

The LCMS/MS analysis of EAF (5 mL) was conducted following the methodology described by Tambunan et al. [40]. A sample extract (5 *μ*L) was separated on an ACQUITY UPLC BEH C18 column (1.7 m × 2.1 mm × 50 mm) at 40°C. A gradient elution technique was employed with 0.1% acetonitrile-formic acid as solvent B flowing at 0.3 mL/min and water formic acid at 0.1% (%v/v) as solvent A in deionised water. The gradient started with a ratio of A:B at 95:5 for the first few minutes, followed by an increase in the proportion of formic acid 0.1% solvent B. The elution gradient was from 5% to 95% B over 30 min. The overall chromatographic run time was 30 min. High-definition mass spectrometry was performed using a XEVO-G2-quadropole (Q)-time of flight mass (ToF) system (Waters, Milford, MA) in V-optics and operated in electrospray ionization (ESI) positive (resolution mode). The optimal analytical conditions included a capillary voltage of 3 kV, a sample cone voltage of 38 V, a desolvation temperature of 300°C, a source temperature of 110°C, a desolvation gas flow of 500 L/h, and a cone gas flow of 16 L/h. The identification of polyphenolic compounds using UPLC-MS/MS was performed by comparing highest mass spectra, retention times, and fragmentation patterns with available spectral databases and available literature data (such as METLIN and MassBank).

#### 2.8.2. HPTLC in Most Bioactive Fractions

The phytochemical quantification of kaempferol, rutin, gallic acid, and quercetin in EAF was carried out using HPTLC, and the results were interpreted by CATS 4 Software. To achieve this, 15 mg of EAF was dissolved in 1 mL of methanol (chromatographic grade) and filtered through Whatman filter paper No. 1. Individual solutions of rutin, kaempferol, gallic acid, and quercetin were prepared in methanol at a concentration of 1 mg/mL. Various solvent combinations were tested for TLC, each fraction, to enhance resolution and maximize spot detection in the EAF. The specific mobile phase compositions for achieving separation were as follows: gallic acid (toluene: ethyl acetate: formic acid: methanol, 5:3:1:0.5); rutin (butanol: acetic acid: water, 4:1:5); quercetin (toluene: ethyl acetate: formic acid, 5:4:0.2); and kaempferol (toluene: ethyl acetate: formic acid: methanol, 5:3:1:5:0.5). Subsequently, each fraction sample (3 *μ*L) was applied to a TLC plate, and a calibration curve was generated using standard markers (quercetin, gallic acid, kaempferol, and rutin) with concentrations ranging from 1 to 8 *μ*g/mL. Initial spot visualization was performed at wavelengths of 254 and 366 nm before derivatization. *R*_*f*_ values and the fingerprint profile photo for the resolved bands at 254 and 366 nm were recorded using WIN-CATS software [41].

### 2.9. Statistical Analysis

The results were presented as the mean ± SEM. To assess significance (*p* < 0.05), Tukey's one-way analysis of variance (ANOVA) was conducted. Different letters denote statistically significant values, whereas identical letters indicate nonsignificant values.

## 3. Result

### 3.1. Yield Percentage

The present study observed that the extraction yield of *E. annuus* CE and its fractions varied. The leaves of *E. annuus* were subjected to methanol extraction, and the resulting CEs were subsequently fractionated using different solvents, selected based on their increasing relative polarity, as indicated by their respective codes in Table 1. Notably, our analysis revealed that the EAF fraction of the *E. annuus* exhibited the highest yield among the various fractions, amounting to 22%. Following this, the AF and CF extracts demonstrated subsequent yields conditions, and temperature can collectively contribute to variations in yield percentage.

### 3.2. Qualitative Analysis of Phytocompounds in Various Extracts of *E. annuus*

The phytochemical analysis of the crude methanolic extract of *E. annuus* leaves, and its fractions demonstrated the presence of different phytoconstituents in all solvents as shown in Table 2.

### 3.3. Quantitative Analysis of Phytoconstituents in Various Extracts of *E. annuus*

**Figure 2:**
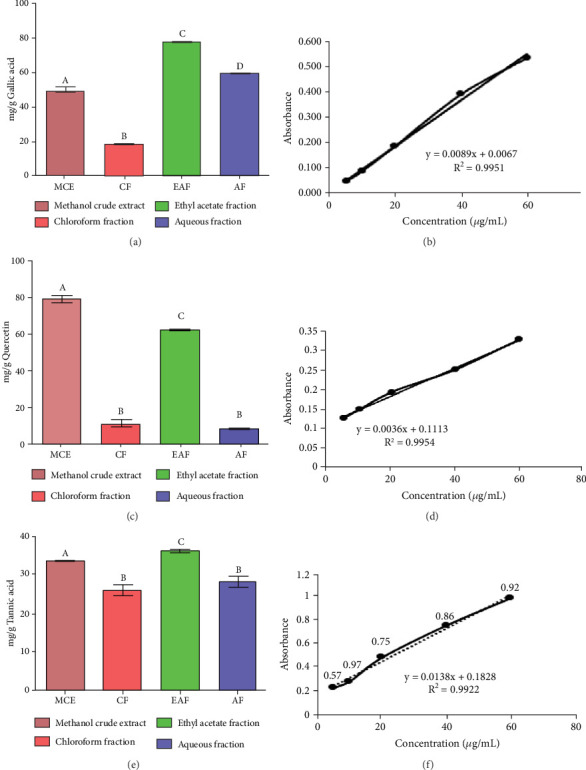
(a) Total phenol content was expressed in mg of gallic acid per gram of crude extract and fractions (dry weight). (b) Standard calibration curve for total phenolic content for standard gallic acid for crude extract and fraction. (c) Total flavonoid content of crude extract and fractions. (d) Standard calibration curve for total flavonoid content for standard quercetin for crude extract and fractions. (e) Total tannin content of all samples. (f) Standard calibration curve for total tannin content for standard tannic acid for crude extract and fractions. The data is presented as mean ± SD. Different superscripts (a–d) on bars are showing significant and nonsignificant variation (analyzed by two-way ANOVA) among crude extract and fraction. Different subscripts indicate significant variation, and the same subscript indicates nonsignificant difference. Error bars are indicated in black color. MCE: methanolic crude extract, CF: chloroform fraction, EAF: ethyl acetate fraction, AF: aqueous fraction.

The leaves of *E. annuus* were subjected to evaluation with CE and fractions for the quantitative estimation of phytochemicals. Several polar compounds in the plant material may account for these findings, as these compounds can dissolve in polar solvents like water, methanol, and ethyl acetate [42, 43]. The total phenolic content (TPC) was notably and significantly (*p* < 0.05) highest in the EAF of *E. annuus* among all the fractions and CE tested. The quantitative assessment of TPC in plant extracts from the leaves of *E. annuus* is illustrated in Figure 2. The determination of TPC content utilized the standard curve of gallic acid, where the linear regression line was presented by the equation *y* = 0.0036*x* + 0.1113, with an *r*^2^ value of 0.9954 as shown Figure 2. The findings indicate that the EAF of *E. annuus* exhibited the highest % of phenols (77.29 ± 0.11 mg/g).

**Figure 3:**
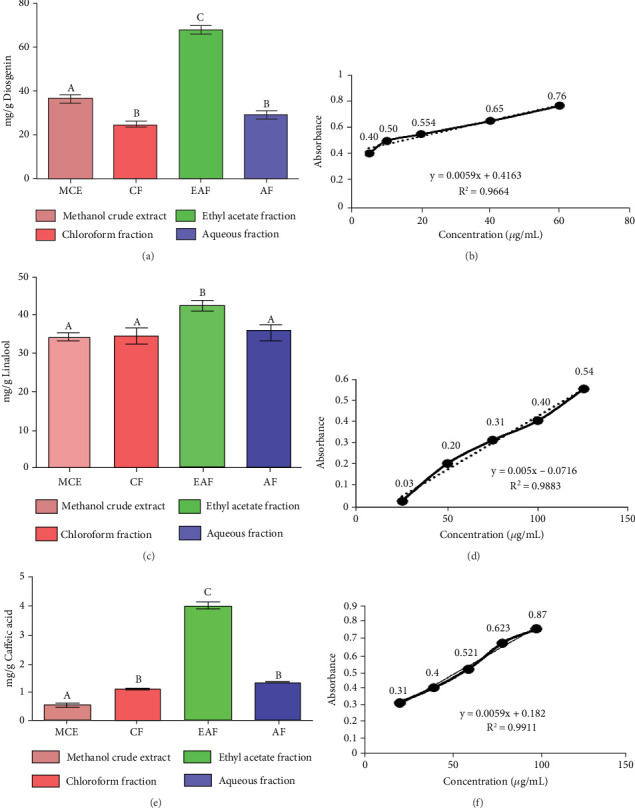
(a) Total saponin content of crude extract and fractions. (b) Standard calibration curve for total saponin content for standard diosgenin for crude extract and fraction. (c) Total terpenoid content. (d) Standard calibration curve for total terpenoid content for standard linalool for crude extract and fractions. (e) Total alkaloid content. (f) Standard calibration curve for total alkaloid content for standard caffeic acid for all sample. The data is presented as mean ± SD. Different superscripts (a–d) on bars are showing significant and non-significant variation (analyzed by two-way ANOVA) among crude extract and fraction. Different subscripts indicate significant variation, and same subscript indicates non-significant difference. Error bars are indicated in black color. MCE: methanolic crude extract, CF: chloroform fraction, EAF: ethyl acetate fraction, AF: aqueous fraction.

TFC was expressed as mg of quercetin equivalent (QUE) per gram of extract (dry weight) (Figure 2c. The results revealed that, among all the solvents tested, the EAF of *E. annuus* displayed the significantly (*p* < 0.05) highest content of flavonoids (80.12 ± 0.21 mg/g). In contrast, the chloroform fraction exhibited the least amount of TFC (7.44 ± 0.11 mg/g). The calibration curve equation for TFC determination was *y* = 0.0089*x* − 0.0067, with an *r*^2^ value of 0.9951 as shown in Figure 2d. The study also demonstrated significant variations among all the fractions. The phytochemical analysis of *E. annuus* revealed notable differences in values among all the fractions employed. Additionally, the study found that, among all the fraction and CE, the EAF of *E. annuus* exhibited the highest concentrations of tannins (Figure 2e. The quantification of tannin components utilized the regression equation derived from the calibration curve: *y* = 0.0138*x* + 0.1828, with an *r*^2^ of 0.9922 as shown in Figure 2f. Among all the fraction and CE, the EAF of *E. annuus* exhibited the highest concentrations of terpenoids as shown in Figure 3a. Terpenoids were quantified using the equation *y* = 0.0059*x* + 0.4163, with an *r*^2^ of 0.9664 as shown in Figure 3b. Similarly, total saponin content was found highest in EAF as shown in Figure 3c. Saponins were quantified using the equation *y* = 0.0053*x* − 0.129, with an *r*^2^ of 0.9883 Figure 3d, and total alkaloid content was found highest found in EAF as shown in Figure 3e. Alkaloids were quantified using the equation *y* = 0.1437*x* + 0.1362, with an *r*^2^ of 0.9911 as shown in Figure 3f. Overall, the study concluded that the EAF from *E. annuus* possessed the highest concentration of various phytoconstituents. Our study suggests that utilizing EAF and AF as solvents produces optimal results in extracting bioactive components from this plant.

### 3.4. DPPH Assay

**Figure 4:**
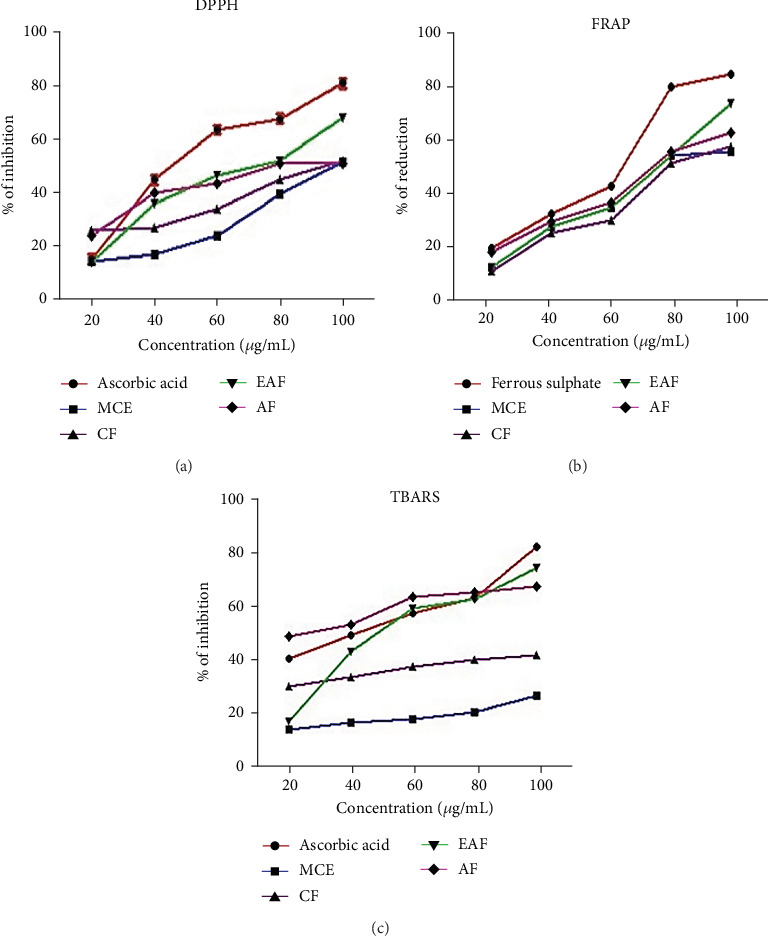
(a) Antioxidant activity represented as the % age of DPPH inhibition. (b) Antioxidant activity using FRAP assay. (c) Inhibition of the production of thiobarbituric acid-reactive substances (TBARS). MCE: methanolic crude extract, CF: chloroform fraction, EAF: ethyl acetate fraction, AF: aqueous fraction.

This study examined the extracts and fractions for their antioxidant activity using DPPH scavenging activity. Antioxidants are crucial in scavenging free radicals, potentially safeguarding against oxidative stress and cell damage [44]. The CE and fractions were compared with ascorbic acid for antioxidant activity, which is recognized for its ability to suppress free radicals. This comparison was assessed using the percentage inhibition depicted in Figure 4, and the IC_50_ values presented in Table 3. The results (Figure 4) revealed a range of % inhibition varying from 22.2 ± 0.1 to 70.1 ± 0.11 in the EAF of *E. annuus_,_* compared with ascorbic acid, which ranged from 13.2 ± 0.2 to 91.1 ± 0.1. Significantly (*p* < 0.05) higher % inhibition was observed in the EAF of *E. annuus* compared with CE and other fractions, possibly attributable to the higher amounts of TPC and TFC in EAF [45].

### 3.5. FRAP Assay

The range of % inhibition in the FRAP assay of EAE of *E. annuus* ranged from 17.43 ± 1.01 to 77.20 ± 0.78, compared with ferrous sulfate, which ranged from 10.25 ± 0.12 to 97.25 ± 1.3*  μ*M of Fe equivalents, as illustrated in Figure 4b. The IC_50_ values are shown in Table 3. Moreover, our findings indicate that, overall, concerning IC_50_ and % inhibition in antioxidant activity among different fractions, the EAF of *E. annuus* leaves appears to possess the highest antioxidant potential.

### 3.6. Lipid Peroxidation Assay

Oxidative stress is measured by identifying indicators related to lipid oxidation. The assessment of lipid peroxidation involves the measurement of malondialdehyde (MDA), the ultimate product of lipid peroxidation. Szychta et al. [46] observed that MDA accurately reflects cellular antioxidant levels. The lipid peroxidation assay results for *E. annuus* extract and its fractions, presented as the % inhibition in Figure 4, revealed that the EAF of *E. annuus* exhibited the highest % inhibition ranging from 30.28 ± 0.38 to 83.87 ± 0.31. As a positive control, ascorbic acid displayed inhibition percentages ranging from 19.26 ± 0.1 to 91.18 ± 0.11. The corresponding IC_50_ values are provided in Table 3.

### 3.7. Glucose Uptake Assay

**Figure 5:**
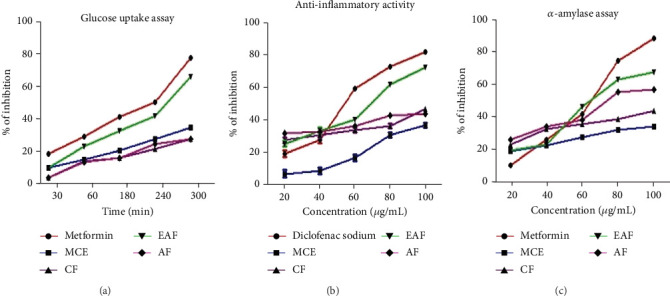
(a) Effect of *Erigeron annuus* crude extract and fractions on the diffusion of glucose out of dialysis membrane. (b) Anti-inflammatory inhibition of *Erigeron annuus* using BSA. (c) Enzyme inhibitory activity of different extracts of *Erigeron annuus* leaf on *α*-amylase. MCE: methanolic crude extract, CF: chloroform fraction, EAF: ethyl acetate fraction, AF: aqueous fraction.

The study examined CE and fractions from *E. annuus* leaves to assess their potential to ameliorate elevated glucose levels. The IC_50_ values for the glucose uptake assay of various herb extracts are shown in Table 3, while the range of % inhibition is depicted in Figure 5a. The results of the glucose uptake assay for *E. annuus* extract and its fractions, expressed as a % inhibition, revealed the EAF fraction of *E. annuus* showed significant activity (ranging from 10.20 ± 0.05 to 66.25 ± 0.07) exhibited a higher % of inhibition compared with other fractions. Metformin, employed as a positive control, displayed inhibition percentages ranging from 18.60 ± 0.01 to 81.25 ± 0.06. These findings suggest that EAF of *E. annuus.* Followed by other fractions, it demonstrates significant (*p* < 0.05) efficacy in quenching glucose molecules and plays a role in alleviating high glucose concentrations.

### 3.8. BSA Protein Denaturation Assay

Protein denaturation refers to the structural loss of proteins' tertiary and secondary configurations, resulting from disrupting and breaking hydrogen, electrostatic, covalent, and disulfide bonds induced by chemical and physical agents [47]. Furthermore, protein denaturation is recognized as a significant contributor to inflammation and inflammatory disorders such as rheumatoid arthritis, diabetes, and cancer [48]. The IC_50_ values of the anti-inflammatory activity of *E. annuus* leaves extracts are shown in Table 3, and the corresponding range of % inhibition is illustrated in Figure 5. The results of the glucose uptake assay for *E. annuus* leaves CE and its fractions presented as the % inhibition, which revealed that the EAF of *E. annuus* (ranging from 22.64 ± 0.24 to 67.23 ± 0.22) exhibited the most potential anti-inflammatory activity against induced protein denaturation, surpassing the standard drug diclofenac sodium (with inhibition percentages ranging from 15.26 ± 0.1 to 92.28 ± 0.1).

### 3.9. *α*-Amylase Inhibition Assay

The findings revealed that the EAF of *E. annuus* showed a noteworthy % of inhibition in the *α*-amylase assay. The results of the *α*-amylase inhibition assay are illustrated in Figure 5c, with the corresponding IC_50_ values provided in Table 3. Notably, the range of inhibition for the EAF of *E. annuus* varied from 20.45 ± 67.30 ± 1. comparing favorably to metformin's range of 19.6 ± 0.05 to 87.9 ± 0.02. All fractions exhibited diverse effects on glucose utilization. Across all concentrations, EAF of *E. annuus* demonstrated a significant (*p* < 0.05) maximal inhibition of the enzyme, reaching the highest value of 67% at a concentration of 100 *μ*g/mL of the plant extract.

### 3.10. *In Vitro* Antiproliferative Activity of the Most Bioactive EAF of *E. annuus* Against the HEK293 Cell Line

**Figure 6:**
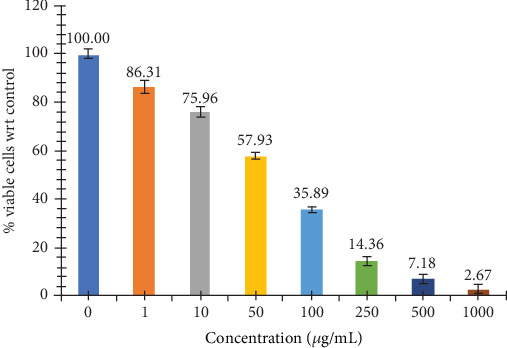
*In vitro* cell cytotoxicity assays of the most bioactive fraction of *Erigeron annuus* (L.) Pers. were performed using the HEK293 cell line. Different doses of ethyl acetate fraction (EAF) of *E. annuus* (0 is control; 1, 10, 50, 100, 250, 500, and 1000 *μ*g/mL is plant concentration) with and without drugs. Data is represented as mean ± SD, and *n* = 6.

**Figure 7:**
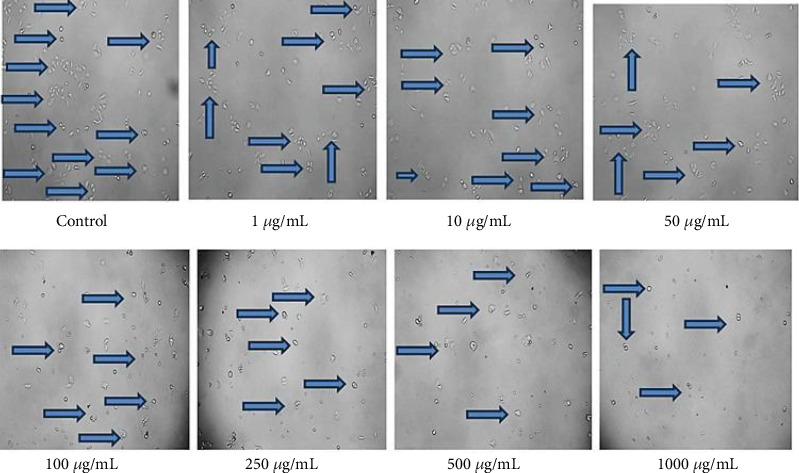
Morphological changes (40x) in HEK293 cells exposed to different concentrations in the most bioactive ethyl acetate fraction (EAF) of *Erigeron annuus* (L.) Pers.

The assessment of EAF of *E. annuus* for potential cytotoxicity is considered as an important in evaluating their suitability for further applications. The HEK293 cell line is originated from the kidney; the kidney nephropathy due to diabetes is very difficult to recover; therefore, we chose this cell to check that at which concentration our plant is nontoxic. Toxicity in HEK293 cells was analyzed by measuring the cell viability using MTT assay. The viability of HEK293 cells exposed 1 to 1000 *μ*g/mL of EAF of *E. annuus* in cell culture. HEK293 cells viability significantly decreased in groups exposed to 250, 500, and 1000 *μ*g/mL of EAF of *E. annuus* extract compared with the control group. The increasing concentration of plant extract decreasing the growth of cell. Cytotoxic potential of EAF of *E. annuus* extract leaves was nontoxic below 50 *μ*g/mL. The cell viability remained above 50% at a concentration of 50 *μ*g/mL, indicating that the plant is nontoxic at this concentration. Different concentrations were taken to check the cell death in cancer cells in different duration of time. MTT assay results suggested that treatment with plant extract significantly reduced the cell viability of cells in a dose-dependent manner as shown in Figure 6. The best results of cell viability of the HEK293 cell line were observed at concentration of 10 *μ*g/mL and 50 *μ*g/mL, because above 50% cell is viable, which mean the plant is not toxic at this concentration after 24 h. Morphological changes were observed using inverted microscope at 40x in HEK293 cells exposed to different concentrations of EAF of *E. annuus,* is shown in Figure 7. Based on the results obtained from the MTT assay, it was observed that when the HEK293 cell line was exposed to different concentrations of the sample, and cytotoxic activity was estimated for samples and 50% inhibitory concentration.

### 3.11. LC-MS/MS Analysis of Most Bioactive Fractions of EAF of *E. annuus*

**Figure 8:**
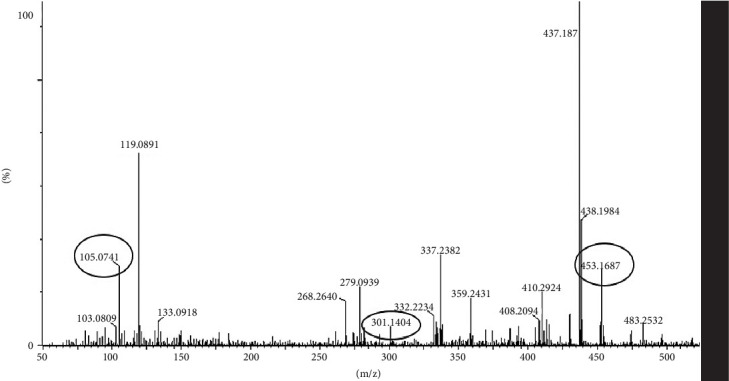
LC-MS/MS spectra of benzoquinone (m/z 105.07), dapdiamide (m/z 301.14), and demethyl-phylloquinone (m/z 453.16) phytoconstituents in the in most bioactive ethyl acetate fraction (EAF) of *Erigeron annuus* (L.) Pers.

**Figure 9:**
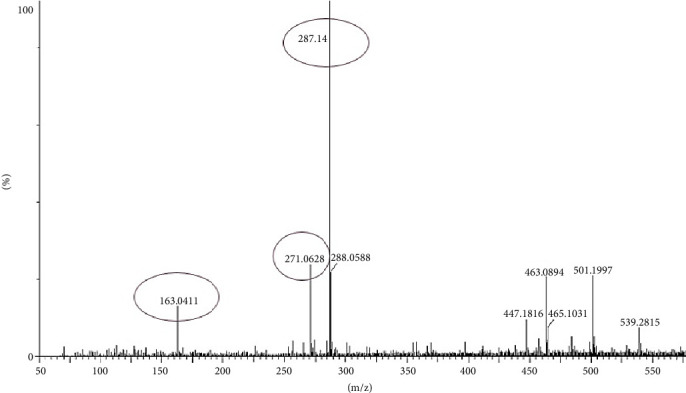
LC-MS/MS spectra of dodecane (m/z 163.04), 2,4⁣′,7-trihydroxyiso-flavanone (m/z 287), retinol (m/z 288.05), and kaempferol and lutinol (m/z 287.14) phytoconstituents in the most bioactive ethyl acetate fraction (EAF) of *Erigeron annuus* (L.) Pers.

LC-MS analysis was performed to identify all non-volatile compounds present in the EAF of *E. annuus*, providing a comprehensive phytochemical profile of the bioactive constituents. A comprehensive LC-MS/MS analysis was conducted to identify the major phytoconstituents in *E. annuus* leaves. The LCMS analysis of the EAF of *E. annuus* revealed a diverse range of compounds with distinct molecular weights. The LCMS scan exhibited various compounds with a retention time (RT) of 2–30 min. The significance of these main compounds lies in their potential roles as antidiabetic agents. The classification of these compounds is based on RT, peak area, m/z ratio, abundance of peaks, and published literature. Distinct peaks were observed at different retention times, with the lowest RT at 2.40 min. The compounds were elucidated based on their molecular weight, with the highest peak at the RT serving as a key identifier, as outlined in Table 4. The LC/MS fragmentation patterns were compared with published literature for accurate identification. The antidiabetic and antioxidant activities of the identified phytochemicals were evaluated based on extensive literature review and published studies. Table 4 shows that the EAF of *E. annuus* leaves contains more flavonoids, with one peak classified as kaempferol. As demonstrated in Figures 8 and 9, kaempferol was a prominent component in the EAF of *E. annuus* leaves bases on spetra peak.

**Figure 10:**
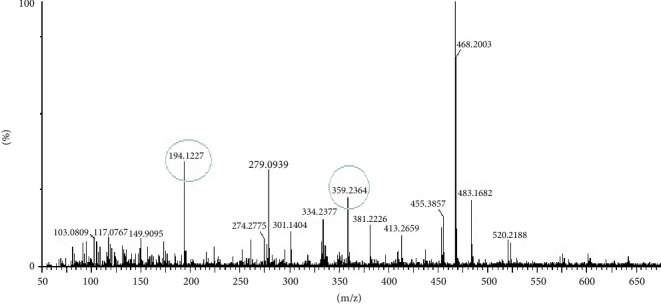
LC-MS/MS spectra of 3,4-O-dimethylgallic acid (m/z 194.122) and 3⁣′,4⁣′,5,7-tetramethylquercetin (m/z 359.23) phytoconstituents in the most bioactive ethyl acetate fraction (EAF) of *Erigeron annuus* (L.) Pers.

**Figure 11:**
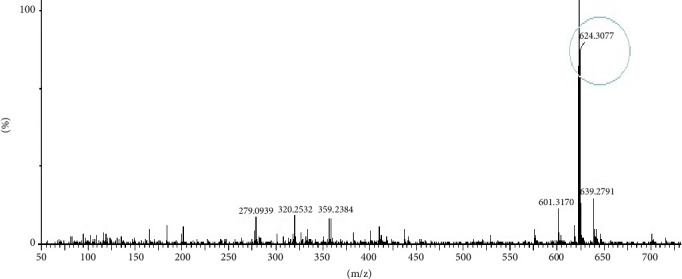
LC-MS/MS spectra of rutin acetate (m/z 624.30) phytoconstituents in the most bioactive ethyl acetate fraction (EAF) of *Erigeron annuus* (L.) Pers.

The compounds present in the sample contribute to their antioxidant and antidiabetic activities. These compounds, namely, dodecane, kaempferol, luteolin, demethylphylloquinone, dapdiamide C, 2,4⁣′,7-trihydroxyiso-flavanone, retinol, and benzoquinone, were subjected to fragmentation, resulting in different fragmentation spectra with observed mass (m/z). LC-MS analysis may not have detected the gallic acid, rutin, and quercetin compounds, but their respective derivatives, namely, 3,4-O-dimethylgallic acid (m/z-194.12, 3⁣′,4⁣′,5,7-tetramethylquercetin (m/z-359.24) as shown in Figure 10 and rutin acetate (m/z-624.30) was identified and their peak is shown in and Figure 11, with their corresponding retention times and m/z values listed in Table 4. The LC-MS/MS analysis of the EAF revealed eight prominent peaks at retention times of 12.17, 17.20, 19.11.10, 21.29, 22.44, 25.54, 34.84, and 36.56 min. The retention times and mass spectra of the different components of the extract were compared with those of authentic samples and mass spectra, as mentioned in Table 4.

### 3.12. HPTLC Chromatogram of Standard Compounds in Most Bioactive Fractions of EAF of *E. annuus*

**Figure 12:**
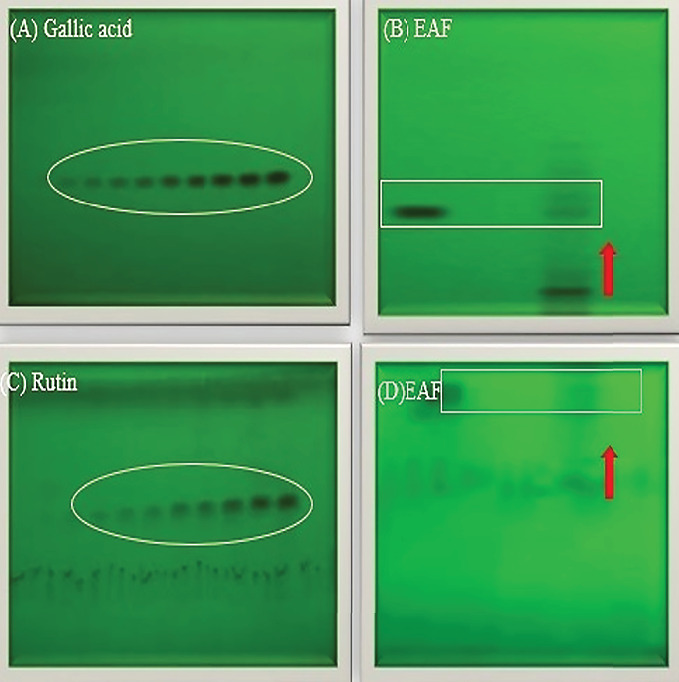
(A) HPTLC chromatogram of standard gallic acid under UV-254 nm. (B) Separation band of gallic acid in most bioactive ethyl acetate fraction. (C) HPTLC chromatogram of standard rutin under UV-254 nm. (D) Separation band of rutin in most bioactive ethyl acetate fractions (EAFs) of *Erigeron annuus* (L.) Pers.

**Figure 13:**
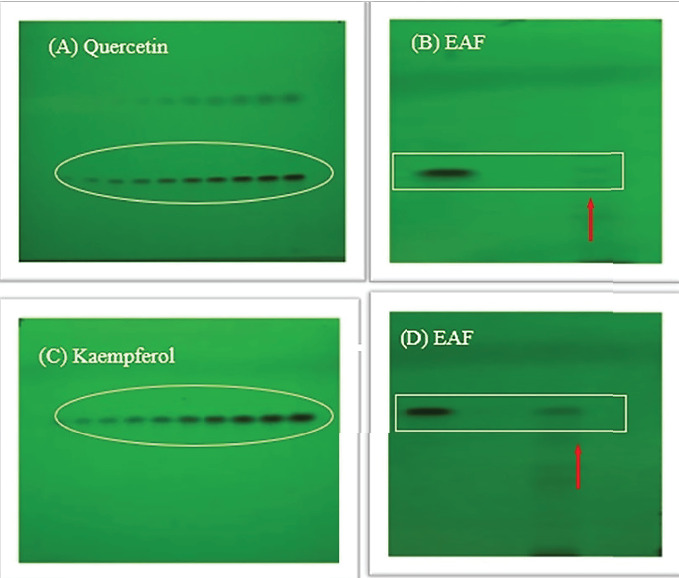
(A) HPTLC chromatogram of standard quercetin under UV-254 nm. (B) Separation band of quercetin in most bioactive ethyl acetate fraction. (C) HPTLC chromatogram of standard kaempferol under UV-254 nm. (D) Separation band of kaempferol in most bioactive ethyl acetate fractions (EAFs) of *Erigeron annuus* (L.) Pers.

In our present investigation, thin layer chromatography was employed to analyze the phytoconstituents within the EAF of *E. annuus*. HPTLC was utilized for the identification of biomarkers, including gallic acid (at 270 nm) and rutin (at 415 nm), as represented in Figures 12A, 12B, 12C, 12D. Additionally, quercetin (at 256 nm) and kaempferol (at 365 nm) in EAF of *E. annuus* extracts were detected at different wavelengths (270 nm), revealing the separation chromatogram of standard compounds alongside bands indicating the separation of plant extracts, as shown in Figures 13A, 13B, 13C, 13D. The quantification of these specific phytochemicals was conducted to assess their contribution to the antidiabetic potential of *E. annuus.*

During the HPTLC analysis, various mobile phases were attempted to separate gallic acid, rutin, quercetin, and kaempferol in different plant extracts. A representative HPTLC chromatogram of standards such as gallic acid, rutin, quercetin, and kaempferol was generated, depicting varying percentage yield s and areas, as well as area percentages, based on different mobile phase compositions. In the EAF of *E. annuus*, the compounds were found in higher percentages, with gallic acid (5.7 *μ*g/mg) leading, followed by rutin (6.11 *μ*g/mg), quercetin 6.1 *μ*g/mg, and kaempferol (7.4 *μ*g/mg) at a retention factor (*R*_*f*_) of 0.49.

## 4. Discussion

Higher percentage extract rates indicate elevated levels of bioactive chemicals in herbal extracts. The variability in yield percentage could be attributed to differences in solvent polarity, which may influence the solubility of the phytoconstituents in the sample and subsequently impact their extraction efficiency. As Mostafa and El-Sayed [42] noted, factors such as chemical composition, solvent polarity, nature of phytoconstituents, types of plant parts, storage conditions, and temperature can collectively contribute to variations in yield percentage. The variations in the polyphenolic content (TPC and TFC) across different fractions could be attributed to the nature of the phytoconstituents, with those more soluble in mid-polar solvents (EAF) than nonpolar solvents (CF) and polar solvents (MCE).

In line with the findings of Kumar et al. [38], who conducted a qualitative study on phytochemical components in different plant extracts, including methanol, chloroform, and petroleum ether extracts, our study demonstrated similar results. Phenols, flavonoids, and saponins were identified as common constituents in all different solvent. Moreover, Rana et al. [48] highlighted the diverse range of phytoconstituents present in *E. annuus*, encompassing alkaloids, flavonoids, saponins, tannins, and terpenoids.

Several polar compounds in the plant material may account for these findings, as these compounds can dissolve in polar solvents like water, methanol, and ethyl acetate [49]. Our study suggests that utilizing EAF as solvents produces optimal results in extracting bioactive components from *E. annuus*. Zhang et al. [43] revealed that the ethyl acetate extract from *E. annuus* leaves contained a total phenolic content of 83.05 ± 0.4*  μ*g/mL and a flavonoid content of 26.2 ± 0.5*  μ*g/mL. Jeong et al. [20] reported that the butanol fraction of *E. annuus* exhibited the highest total phenolic content (396.49 mg of GAE/g), followed by the AF (241.87 mg of GAE/g), and lastly, the chloroform fraction (107.34 mg of GAE/g).

Earlier studies by Lee and Seo [50] demonstrated that the methanol extract of *E. annuus* exhibited a more effective DPPH removal than the water extract. The findings of the present study align with those of previous research. The investigation also revealed variations across all antioxidant assays, potentially attributed to the plant's extracts elevated levels of phenols and flavonoids [51]. Studies suggest that the antioxidant activity of plant associated with their phenolic redox characteristics, serving as reducing agents, singlet oxygen quenchers, and hydrogen donors.

Similar results were observed by Lee and Seo [50] who reported the antioxidant properties of *E. annuus_,_* which were possibly attributed to the presence of phenolic compounds. The ethyl acetate extract (253.2 ± 2.0*  μ*g/mL) exhibited scavenging activity, demonstrating its ability to scavenge free radicals, and this activity was closely linked to the polarity of the *E. annuus* extract. Similarly, Jeong et al. [52] found that the butanol fraction displayed the highest antioxidant activity, as evidenced in the ABTS and FRAP assays. Abd EI-Rahim et al. [51] highlighted the influence of extraction solvents on both extraction yield and the content of bioactive compounds, consequently significantly impacting the biological activity of the extract. Baccheti et al. [53] reported that the methanol extract of *Fagopyrum esculentum* leaves demonstrated potent DPPH radical scavenging effects along with reducing power, with an antioxidant activity of 20.00 ± 2.67*  μ*g/mL. Oxidative stress is measured by identifying indicators related to lipid oxidation. Kim and Choi [54] observed that MDA accurately reflects cellular antioxidant levels.

Diabetes disrupts energy balance and is symptomatically characterized by glucose intolerance, contributing to the diabetic condition. Yoo et al. [55] observed similar outcomes in *Erigeron breviscapus* and noted its ability to enhance glucose uptake. However, the antidiabetic potential of *E. annuus* leaves through *in vitro* glucose uptake assay has not been investigated thus far. This assay is valuable for assessing the capability of drugs to either facilitate or inhibit the entry of glucose into cells.

In a separate investigation, the methanolic extract derived from the roots of *E. annuus* demonstrated a significant reduction in inflammation, ranging from 29% to 50%, with concentrations ranging from 3 to 30 *μ*g/mL [53]. Furthermore, Rana et al. [42] identified various phytochemicals with antidiabetic properties extracted from ethyl acetate extract of *E. annuus* flowers. Notably, there have been no reported adverse effects or signs of toxicity associated with the plant, encouraging researchers to explore developing an appropriate dosage form for administering this plant as a potential treatment for various ailments.

An effective approach to reducing postprandial blood glucose levels involves blocking *α*-amylase activity. Similarly, Jeong et al. [20] highlighted the potential inhibitory effects of various extracts from *E. annuus* flower on *α*-amylase activity. The chloroform extract emerged as the most effective, boasting an IC_50_ value of 0.8 ± 0.0*  μ*g/mL. Following closely were the ethyl acetate (1.1 ± 0.0*  μ*g/mL), hexane (1.2 ± 0.0*  μ*g/mL), and acetone (1.3 ± 0.0*  μ*g/mL) extracts, all surpassing the inhibitory activity of acarbose (2.4 ± 0.0*  μ*g/mL). The ethanol extract (2.4 ± 0.0*  μ*g/mL) exhibited a comparable inhibitory effect to acarbose, while methanol (2.7 ± 0.0*  μ*g/mL) and water (4.2 ± 0.0*  μ*g/mL) extracts demonstrated lower values than acarbose. In addition, Yoo et al. [55] found that flavonoids extracted from the ethyl acetate extract of *E. annuus* flower exhibited inhibitory effects on the production of advanced glycation end-products and the actions of aldose reductase. These findings offer insights into the robust hyperglycemic properties of ethyl acetate extract, suggesting its potential as a valuable resource for developing novel and cost-effective hyperglycemic agents.

Additionally, Nazaruk et al. [56] investigated the ability of essential oils from *Erigeron acris* roots and herb, as well as *Erigeron annuus* herb, to stop cell growth. They performed the cell viability experiment on cultured fibroblasts, cancer cell lines (MCF-7 and MDA-MBA-231), and colon adenocarcinoma (DLD-1) cells, using 3-(4,5-dimethylthiazol-2-yl)-2,5-diphenyltetrazolium bromide. With an IC_50_ value of 14.5 *μ*g/mL, the essential oil from *Erigeron acris* roots had the most potent effect on stopping cell growth in the MCF-7 cell line.

Analysis of the EAF of *E. annuus* using LC-MS revealed variations in the separated compounds, as indicated by different RTs, peak areas, and *m/z* values. The compounds, dodecane, kaempferol, luteolin, demethylphylloquinone, dapdiamide C, 2,4⁣′,7-trihydroxyiso-flavanone, retinol, and benzoquinone comparing molecular weight (M) and m/z fragment with the literature data. Several studies have reported that the above mentioned phytochemicals exhibited both antioxidant and antidiabetic activities [57–65]. Although gallic acid, rutin, and quercetin themselves were not detectable in the LC-MS chromatogram due to the low concentrations of these compounds [66]. Moreover, 3,4-O-dimethylgallic acid (m/z-194.12, 3⁣′,4⁣′,5,7-tetramethylquercetin (m/z-359.24), and rutin acetate (m/z-624.30) exhibited various biological activities [67]. According to Mohammadhosseini et al. [68], the occurrence of a variable pattern in the distribution of compounds may account for the diverse biological activities observed. The chloroform, ethanolic, and ethyl acetate extracts of the aerial parts of *E. annuus* have been documented to contain a range of triterpenes and phytosterols, including *β*-sitosterol glucoside, friedela-3-one, lupeol, oleanolic acid, betulinic acid, *β*-sitosterol, 29-O-*β*-D-glucopyranosyl-3*β*, ursolonic acid, 3*β*,23,28-triol olean-12-ene, 3*β*,23-dihydroxyl-29-O-*β*-D-glucopyranosylolean-12-en-28-oic, hexacetate, and 23-dihydroxyolean-12en-28-oic acid.

HPTLC is an important analytical method for qualitative and quantitative assessment of plant phytochemicals [69]. This includes generating TLC fingerprint profiles and determining chemical markers and biomarkers [40]. Our observation aligns with similar findings by Mehesare et al. [70] who, while working with the hydro-alcoholic seed extract of *Holarrhena antidysenterica*, detected and quantified gallic acid, quercetin, and rutin, noting a higher percentage yield of gallic acid.

## 5. Conclusion

The study reveals that the EAF of *E. annuus* exhibited potential antioxidant property and effectively inhibited the activity of the *α*-amylase enzyme. This inhibition of *α*-amylase enzyme activity opens new possibilities for the development of plant-derived medicinal agents for diabetes. The EAF of *E. annuus* holds significant promise as a potential natural remedy for diabetes. Additionally, LC-MS/MS technique was employed to tentatively identify phytochemicals within the EAF of *E. annuus*. Furthermore, HPTLC was employed to quantify the substances. Further investigations are required to validate these findings and explore the potential application of *E. annuus* in managing insulin resistance in human patients. Ongoing research is essential to identify the key compounds in *E. annuus* and understand their mechanisms of effect on peroxisome proliferator-activated receptors, enhancing insulin effectiveness, targeting tissues and other insulin-related pathways associated with the pathophysiology of diabetes mellitus. Moreover, an *in silico* approach can be employed to kaempferol phytochemical identified through LC-MS or HPTLC, enabling computational evaluation of their biological activity and molecular interactions.

## Figures and Tables

**Table 1:** Description of collected plants, their coding, and corresponding yield percentages.

**Extract of *E. annuus***	**Yield (%)**
MCE	21.08 ± 1.22
CF	12.06 ± 1.09
EAF	22 ± 1.11
AF residue	13.3 ± 1.02

Abbreviations: MCE: methanolic crude extract, CF: chloroform fraction, EAF: ethyl acetate fraction, and AF: aqueous fraction.

The data is presented as mean ± SD.

**Table 2:** Qualitative analysis of phytochemicals from leaf extracts of *E. annuus* in different solvents.

**Phytochemicals**	**MCF**	**CF**	**EAF**	**AF**
Phenols	+	+	++	++
Flavonoids	+	+	+++	+
Tannins	+	+	+++	+
Saponins	++	++	++	+
Terpenoids	+	+	+++	+
Alkaloids	+	+	+	+

*Note:* ‘+++' means highly present, ‘++' means moderately present, ‘+' means present.

Abbreviations: MCE: methanol crude extract, CF: chloroform fraction, EAF: ethyl acetate fraction, AF: aqueous fraction.

**Table 3:** IC_50_ values of *E. annuus* and its fractions.

**Biological activities**	**Extracts/standard**	***E. annuus* (IC**_**50**_ **)**
Antioxidant activity (DPPH) (*μ*g/mL)	MCE	102.65 ± 0.80^a^
	CF	97.36 ± 0.71^b^
	EAF	46.71 ± 0.67^c^
	AF	76.55 ± 0.58^d^
	Ascorbic acid	22.27 ± 0.96^e^

FRAP (*μ*M Fe equivalents)	MCE	63.27 ± 0.38^a^
	CF	83.99 ± 0.12^b^
	EAF	30.43 ± 0.25^c^
	AF	34.31 ± 0.11^d^
	Ferrous sulfate	9.73 ± 0.24^e^

Anti-inflammatory activity (*μ*g/mL)	MCE	110.95 ± 0.78^a^
	CF	98.72 ± 1.71^b^
	EAF	74.79 ± 1.56^c^
	AF	115.74 ± 1.46^d^
	Diclofenac sodium	50.39 ± 0.65^e^

Alpha amyalse activity (*μ*g/mL)	MCE	145.27 ± 0.01^a^
	CF	54.42 ± 0.01^b^
	EAF	40.59 ± 0.03^c^
	AF	54.80 ± 0.88^b^
	Metformin	19.92 ± 0.14^d^

Glucose assay (*μ*g/mL)	MCE	9.16 ± 0.37^b^
	CF	7.62 ± 0.95^a^
	EAF	6.84 ± 1.74^a^
	AF	9.21 ± 1.47^b^
	Metformin	1.94 ± 0.36^d^

TBARS (*μ*g/mL)	MCE	50.31 ± 0.32^a^
	CF	62.29 ± 0.129^b^
	EAF	45.259 ± 0.98^c^
	AF	71.45 ± 0.25^d^
	Ascorbic acid	26.58 ± 0.41^e^

*Note:* Data are expressed as mean ± SD (*n* = 3). The Tukey one-way ANOVA was employed to ascertain the significance level of all plant samples.

^a-n^The same superscript in a column do not significantly differ from each other (*p* < 0.05).

**Table 4:** Identification of key phytoconstituents in the EAF of *E. annuus* using LCMS/MS for their antioxidant and anti-diabetic efficacy.

**Compounds**	**Molecular weight**	**Chemical formula**	**Spectra peak (m/z)**	**Retention time (min)**
Dodecane	170.34	C_12_H_26_	163.04	25.88
Kaempferol	287.05	C_15_H_10_O_6_	287.14	25.63
Luteolin	286.05	C_15_H_10_O_6_	287.14	27.64
Demethyl-phylloquinone	450.34	C_31_H_46_O_2_	453.18	17.20
Dapdiamide	300.33	C_12_H_20_N_4_O_5_	301.14	36.86
2,4⁣′,7-Trihydroxyiso-flavanone	272.25	C_15_H_12_O_5_	271.06	22.44
Retinol	286.46	C_20_H_30_O	288.05	19.11
Benzoquinone	108.09	C_6_H_4_O_2_	105.07	25.54
3,4-O-Dimethylgallic acid	194.03	C_8_H_8_O_5_	194.12	30.40
3⁣′,4⁣′,5,7-Tetramethylquercetin	358.10	C_19_H_18_O_7_	359.24	2.40
Rutin acetate	610.51	C_27_H_30_O	624.30	24.39

## Data Availability

All data produced in this study are enclosed in this research. All figures were created in Software/PowerPoint.
